# Consistent tracer administration profile improves test–retest repeatability of myocardial blood flow quantification with ^82^Rb dynamic PET imaging

**DOI:** 10.1007/s12350-016-0698-6

**Published:** 2016-11-01

**Authors:** Ran Klein, Adrian Ocneanu, Jennifer M. Renaud, Maria C. Ziadi, Rob S. B. Beanlands, Robert A. deKemp

**Affiliations:** 1 National Cardiac PET Centre, Division of Cardiology, Department of Medicine, University of Ottawa Heart Institute, Ottawa, Canada; 2 Department of Systems and Computer Engineering, Carleton University, Ottawa, Canada; 3 Division of Nuclear Medicine, Department of Medicine, The Ottawa Hospital and University of Ottawa, Box 232, 1053 Carling Ave, Ottawa, ON K1Y 4E9 Canada; 4 Present Address: Non Invasive Cardiovascular Imaging Department, Diagnostico Medico Oroño, Rosario, Argentina

**Keywords:** Rubidium-82, myocardial blood flow, reproducibility, square-wave infusion profile

## Abstract

**Objectives:**

Quantification of myocardial blood flow (MBF) and stress/rest flow reserve is used increasingly to diagnose multi-vessel coronary artery disease and micro-vascular disease with PET imaging. However, variability in the measurements may limit physician confidence to direct revascularization therapies based on specific threshold values. This study evaluated the effects of rubidium-82 (^82^Rb) tracer injection profile using a constant-activity-rate (CA) vs a constant-flow-rate (CF) infusion to improve test–retest repeatability of MBF measurements.

**Method:**

22 participants underwent single-session ^82^Rb dynamic PET imaging during rest and dipyridamole stress using one of 2 test–retest infusion protocols: CA–CA (*n* = 12) or CA–CF (*n* = 10). MBF was quantified using a single-tissue-compartment model (1TCM) and a simplified retention model (SRM). Non-parametric test–retest repeatability coefficients (RPC_np_) were compared between groups. Myocardium-to-blood contrast and signal-to-noise ratios of the late uptake images (2 to 6 minutes) were also compared to evaluate standard myocardial perfusion image (MPI) quality.

**Results:**

MBF values in the CA–CA group were more repeatable (smaller RPC_np_) than the CA–CF group using the 1TCM at rest alone, rest and stress combined, and stress/rest reserve (21% vs 36%, 16% vs 19%, and 20% vs 27%, *P* < 0.05, respectively), and using the SRM at Rest and Stress alone, Rest and Stress combined, and stress/rest reserve (21% vs 38%, 15% vs 25%, 22% vs 38%, and 23% vs 49%, *P* < 0.05, respectively). In terms of image quality, myocardium-to-blood contrast and signal-to-noise ratios were not significantly different between groups.

**Conclusions:**

Constant-activity-rate ‘square-wave’ infusion of ^82^Rb produces more repeatable tracer injection profiles and decreases the test–retest variability of MBF measurements, when compared to a constant-flow-rate ‘bolus’ administration of ^82^Rb, especially with SRM, and without compromising standard MPI quality.

**Electronic supplementary material:**

The online version of this article (doi:10.1007/s12350-016-0698-6) contains supplementary material, which is available to authorized users.

## Introduction

Absolute myocardial blood flow (MBF) quantification addresses the limitation of relative myocardial perfusion imaging (MPI) by measuring blood flow in absolute units of mL/min per gram of tissue (mL/min/g), with proven precision and accuracy^1^,^2^ and incremental prognostic value.^3^,^4^ MBF quantification with rubidium-82-chloride (^82^Rb) positron emission tomography (PET) requires minimal changes to the conventional image acquisition protocol, with no additional risk or discomfort to patients. A series of dynamic PET images are acquired starting at the time of tracer administration to measure the time course of tracer clearance from arterial blood and uptake into the myocardium. Tracer kinetic modeling analysis is then used to estimate MBF based on the rate of tracer uptake measured in the dynamic image sequence.

To enable high diagnostic confidence in the MBF measurements, the test–retest repeatability should have low variance, which is dependent on several factors during image acquisition and analysis. We have previously shown that automation of processing helps to minimize operator-induced variability,^5^ and standardized analysis protocols are reproducible between laboratories.^6^ In theory, tracer kinetic analysis should be robust against variations in the shape of tracer injection profiles; however, in practice, the estimated kinetic parameters may be biased due to limitations in instrumentation and modeling assumptions.^7^ There are limited studies investigating the influence of tracer infusion profiles on MBF quantification.^8^,^9^

^82^Rb is the PET tracer used most commonly for clinical MPI, and its use is growing for MBF quantification. Test-retest repeatability of MBF imaging has been reported in the range of 25% to 40% using dipyridamole^10^–^12^ and 25% to 30% using regadenoson stress.^13^ Due to the short radioactive half-life (76 second) of the generator-produced ^82^Rb isotope, the tracer must be eluted directly to the patient through an intravenous (IV) catheter. The shape of the eluted activity profile vs time can vary dramatically over the life of the generator, as the parent ^82^Sr isotope decays. We previously reported the performance of a custom elution system to allow accurate administration of ^82^Rb activity using variable flow rates within a preset time interval, e.g., 30 second ‘slow-bolus’ infusions.^14^ Subsequent developments have aimed to improve the repeatability of tracer infusion profiles that would otherwise change with the age of the ^82^Sr/^82^Rb generator. This infusion system now employs a generator bypass-line and a feedback-control system to achieve two key features: (1) administration of ^82^Rb activity at a constant activity rate (MBq/s) to avoid saturating the PET detectors during dynamic image acquisition, and (2) repeatable ‘square-wave’ ^82^Rb activity profiles with progressive generator aging.

The primary goal of this work was to characterize the influence of variable tracer infusion profiles on the test–retest repeatability of MBF quantification, using two common tracer kinetic models: (1) the single-tissue-compartment model (1TCM)^15^ and (2) a simplified retention model (SRM).^16^,^17^ A secondary goal was to evaluate the effect of tracer infusion profiles on standard MPI signal-to-noise and contrast as measures of image quality. We hypothesized that MBF estimates may be dependent on the shape of the tracer infusion profiles, and that the test–retest variability of MBF quantification may be reduced by infusing ^82^Rb at a constant activity rate (CA) vs a constant flow rate (CF), without a significant impact on standard perfusion image quality.

## Materials and Methods

### Study Population

This study consisted initially of 24 participants: 15 clinical patients with known or suspected coronary artery disease (CAD) and 9 healthy subjects with low risk of CAD.^18^ Patients with acute coronary syndrome or unstable angina, heart failure, pulmonary edema, severe valve disease, or contraindication to dipyridamole such as hypotension, heart block, or asthma were excluded. One healthy subject was excluded due to interstitial tracer injections resulting from poor IV cannulation, and one clinical patient was excluded due to paced rhythm that was discovered following enrolment. Subjects were instructed to abstain from caffeine intake for 12 hours, fast for 4 hours (except for water intake), and withhold cardiac medications prior to the study, according to our clinical protocol and society guidelines.^19^ All participants provided written informed consent to participate under a research protocol approved by the University of Ottawa Heart Institute Human Research Ethics Board.

### Image Acquisition

Our standard clinical imaging protocol^20^ was modified to acquire two rest and two stress (test–retest) scans in a single imaging session, to maintain consistent patient positioning and hemodynamic conditions. Patients were positioned in a Discovery 690 PET/VCT-64 scanner (GE Healthcare, Waukesha, WI) with ECG leads placed for patient monitoring and cardiac gating. A scout scan was performed for patient positioning, followed by a low-dose [0.14 to 0.37 mSv] (median 0.26 mSv) CT scan for attenuation correction. Four list-mode PET scans were acquired in 3D-mode; two scans at rest and two during dipyridamole-induced hyperemic stress. ^82^Rb was administered over a 30-second interval as a standard ‘square-wave’ infusion using the CA elution mode for the first (test) rest and stress scans, according to our routine clinical practice (Ruby-Fill^®^ generator and prototype Rb-82 elution system [v2], Jubilant DraxImage, Kirkland, QC).^20^ For the second (retest) rest and stress scans, either the same CA infusion or a 30 mL/min CF ‘bolus’ infusion mode was used.

For all scans, the injected activity was adjusted for patient weight (10 MBq/kg) to limit the scanner coincidence dead-time to <35% and the corresponding dead-time correction factors (DTF < 1.54) to ensure accurate measurement of the bolus first-pass activity.^21^ ^82^Rb PET images were aligned with the CT images for accurate attenuation correction prior to dynamic image reconstruction. List-mode scans (6 minute) were rebinned into 14 time frames (9 × 10, 3 × 30, 1 × 60, 1 × 120 seconds) and reconstructed using the vendor iterative algorithm (OSEM 24 subsets, 4 iterations) and 8 mm Hann post-filter.

### Tracer Infusion

**Figure 1:**
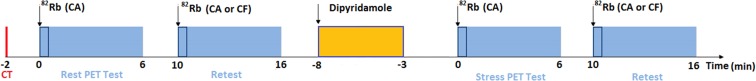
Test–retest ^82^Rb PET imaging protocol. A 30 seconds constant-activity-rate (CA) ‘square-wave’ infusion was used for both test–retest scans in the CA–CA cohort, whereas a 30 mL/min constant-flow-rate (CF) ‘bolus’ infusion was used for the retest scans in the CA–CF cohort

Participants were randomly assigned to one of two groups to avoid selection bias. In the CA–CA group, all four scans (test and retest at rest and stress) were performed using the CA infusion mode. In the CA–CF group, imaging was performed using the CA infusion mode for the first (test) rest and stress scans, and the CF infusion mode for the second (retest) rest and stress scans. CA infusions were always performed first to conform to our standard clinical imaging protocol. CF infusions were performed at the maximum flow rate of 30 mL/min, to achieve the shortest possible bolus infusion. All scans were initiated manually after ^82^Rb infusion was started, and the scanner-reported coincidence (prompt) count rates exceeded 10 kcps. The first rest scan was followed immediately by a second rest scan. The stress agent, dipyridamole (0.14 mg/kg/min), was infused for 5, and 3 minutes later, the two stress scans were performed in rapid succession, as illustrated in Figure 1.

### Image Quality

Since MPI is currently the standard for clinical interpretation, the effect of CA vs CF infusion profiles on image quality was also evaluated. Several metrics were utilized: the total coincidence (prompt) counts recorded during the 2 to 6 minutes retention phase, the left ventricle (LV) activity polar map myocardium-to-blood ratio (MBR), contrast-to-noise ratio (CNR), and the myocardium signal-to-noise ratio (SNR). LV activity polar maps were sampled from the 2 to 6 minutes retention-phase images using FlowQuant^®^ V2.4 (UOHI, Ottawa, ON). MBR was defined as the mean tracer activity in the LV polar map divided by the arterial blood value during the same time frames (2 to 6 minutes). A higher MBR value indicates a higher retention of radioactive tracer in the myocardium with respect to the residual activity in the blood. A narrower peak of the bolus first-pass in the blood input using the CF elution mode might improve the MBR due to longer blood clearance time, but may also increase the PET detector dead-time losses; therefore, the peak dead-time correction factor (DTF) was also recorded. SNR was computed as the mean/standard deviation (SD) of the myocardium activity polar map. CNR was defined as the mean (myocardium—blood) contrast divided by the SD of the myocardium value in the LV activity polar map. Higher CNR and SNR are indicative of lower image noise and higher uniformity of tracer retention in the myocardium.

### Myocardial Blood Flow

Reconstructed dynamic PET image sequences were analyzed with FlowQuant^®^ to quantify MBF in the 3 vascular territories using the 1TCM with dual-spillover correction and tracer extraction correction, previously shown to have good test–retest repeatability at rest.^10^ This method includes automatically derived left ventricle orientation and segmentation with optional operator adjustments. The arterial blood volume of interest was automatically derived by thresholding segmentation of the peak-activity blood pool image in a restricted region including the left atrium and aortic outflow tract as described previously.^10^ A second kinetic model, the SRM was also used to quantify MBF using the same regions described above, as reported previously using a fixed recovery coefficient (RC = 0.76), a blood integration interval from time zero to the blood peak-time + 1.4 minutes, and an extraction correction consistent with the 1TCM values of MBF.^17^

## Statistical Analysis

Continuous and discrete data are presented as mean ± standard deviation (SD) and range [minimum, maximum] or median and inter-quartile range (IQR) for non-Gaussian-distributed data. Demographic and hemodynamic variables were compared using unpaired or paired Student’s t-tests, with Bonferroni corrections as appropriate. test–retest MBF values were compared using Spearman’s rank correlation (R). Differences in repeated measurements were calculated both in absolute units (retest–test) mL/min/g, and relative to the test–retest mean values [(retest − test)/(retest + test)/2 × 100%]. The measured test–retest MBF differences did not follow a Gaussian distribution so non-parametric repeatability coefficients (RPC_np_=1.45×IQR) were used as a more robust measure to characterize the repeatability. For Gaussian-distributed data, the conventional RPC=1.96×SD and RPC_np_ are equivalent.^22^ To account for small measured differences in the test–retest values measured separately at Rest and Stress, repeatability of the combined Stress & Rest data was assessed using values adjusted for the median differences. Changes in the heart rate × systolic blood pressure = rate × pressure product (RPP) between test and retest were compared to differences in MBF using Spearman’s rank correlation. Wilcoxon and Levene’s non-parametric tests were used to assess the statistical significance of differences in medians and variances, respectively. *P* values less than 0.05 were considered statistically significant. All analyses were performed using Matlab R2013b (Mathworks, Natick, MA).

## Results

### Study Population

Demographics and cardiac risk factors for the patients and normal volunteers are summarized in Table 1. The CA–CF cohort had a higher number of healthy normals who tended to be younger with lower BMI than the CA–CA subjects. Hemodynamic measurements are summarized in Table 2. As expected during pharmacologic stress, there were significant increases in HR, BP, and RPP vs the resting state values. There were no changes in resting hemodynamics between test and retest; however, at stress, there was a small decrease observed in all the hemodynamic values during the retest scans compared to the initial stress test scans (*P* < 0.05).

**Table 1:** Demographics and cardiac risk factors

	CA–CA cohort	CA–CF cohort	*P* value
Total subjects (*n*)	12	10	
Healthy normals (*n*)	2	6	0.04
Sex (male)	6	3	0.34
Age (mean ± SD [range]) years	62.2 ± 9.6 [47, 81]	54.3 ± 12.3 [25, 67]	0.11
BMI (mean ± SD [range]) m^2^/kg	32.5 ± 6.1 [24, 43]	28.1 ± 4.2 [22, 34]	0.06
Diabetic (No/Type 1 DM/Type 2 DM)	10/1/1	10/0/0	0.2/0.4/0.4
Smoker (Never/current/past >1 year)	6/3/3	9/0/1	0.4/0.1/0.4
Single vessel disease (*n*)	3	1	0.36
Multi-vessel disease (*n*)	1	0	0.40
LV ejection fraction at rest (%)	55 ± 9	62 ± 7	0.06
LV ejection fraction at stress (%)	64 ± 10	70 ± 3	0.06

*BMI* body mass index (height^2^/weight)

*Type 1 DM* insulin-dependent diabetes mellitus

*Type 2 DM* non-insulin-dependent diabetes mellitus

**Table 2:** Hemodynamic parameters (mean ± SD)

*n* = 22	Rest		Stress	
	Test	Retest	Test	Retest
HR (bpm)	66.3 ± 9.7	65.2 ± 9.0	92.1 ± 14.2*	85.5 ± 11.3*^†^
Systolic BP (mmHg)	129.9 ± 19.2	128.4 ± 17.6	137.8 ± 23.3*	129.5 ± 19.5^†^
Diastolic BP (mmHg)	76.0 ± 8.9	75.1 ± 7.3	82.3 ± 13.8*	73.9 ± 10.3^†^
RPP (bpm × mmHg)	8700 ± 2263	8435 ± 1995	12769 ± 3309*	11162 ± 2648*^†^

*HR* heart rate, *BP* blood pressure, *RPP* rate pressure product (HR × systolic BP), *bpm* beats per minute, *mmHg* millimeters of mercury

* *P* < 0.05 increase during stress vs rest

^†^ *P* < 0.05 decrease during retest vs test

**Figure 2:**
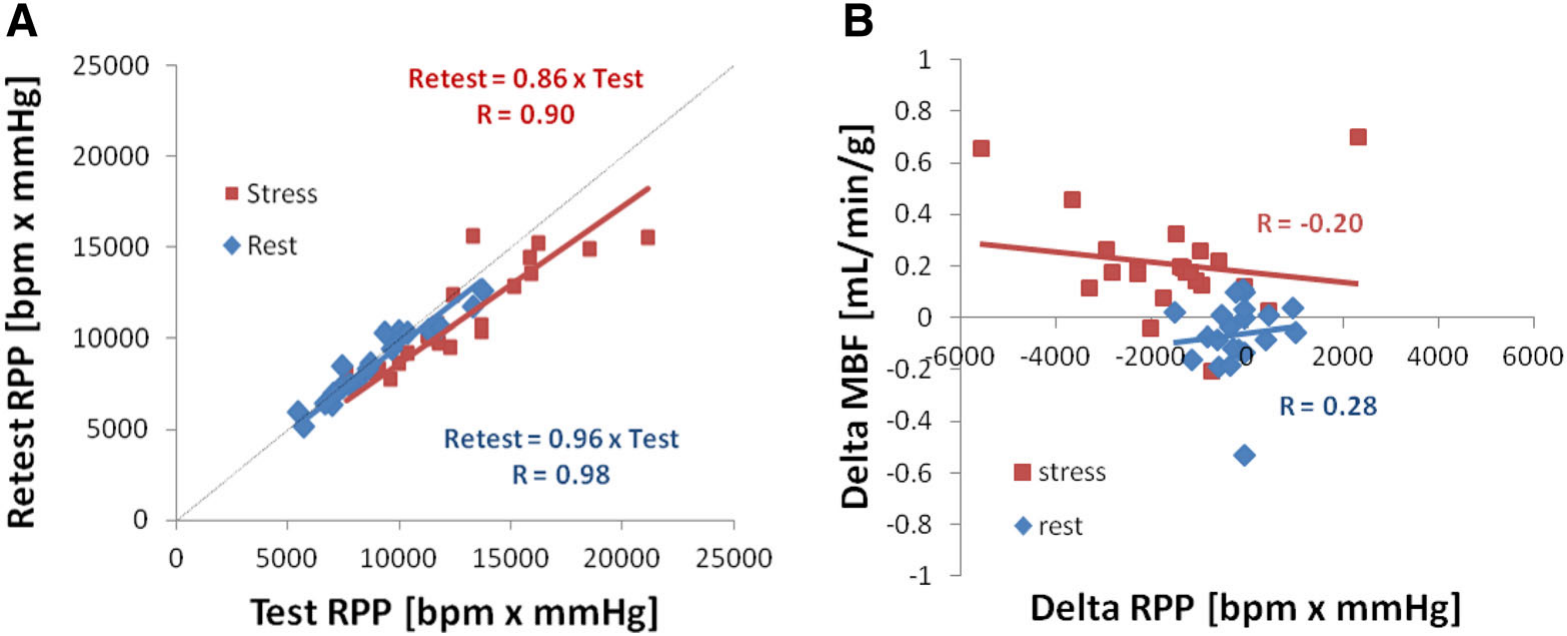
Hemodynamic measurements during test and retest scans. (**A**) Very good correlation of retest vs test heart rate × systolic blood-pressure product (RPP) at rest and stress, and (**B**) no significant correlation of test–retest (Delta) changes in MBF (using 1TCM) vs changes in RPP at rest or stress

Retest vs test RPP values were highly correlated (*R* ≥ 0.90; *P* < 0.001), showing a small but significant decrease both at rest and stress (Figure 2A). The test–retest changes in MBF (Delta) were not significantly correlated with changes in RPP (*R* < 0.30; *P* = NS) as shown in Figure 2B using 1TCM, nor with SRM (data not shown), therefore no RPP-adjustments of rest or stress MBF values were performed.

### Tracer Infusion

**Figure 3:**
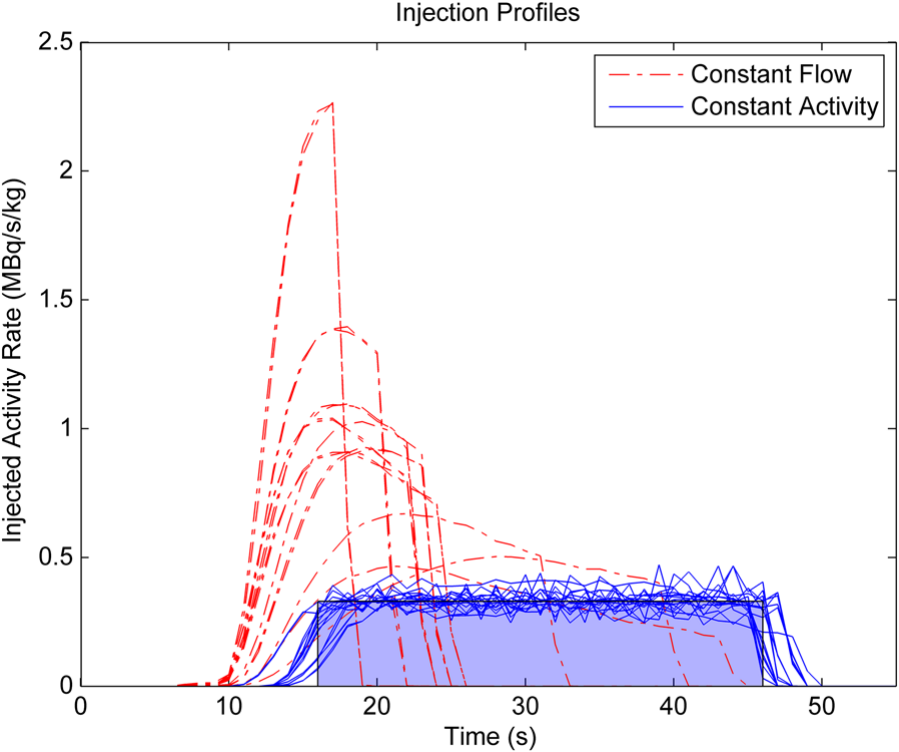
^82^Rb infusion profiles for the CA–CF test–retest cohort. The CA-mode elution profiles follow an approximate ‘square-wave’ infusion profile with a constant activity rate of 0.33 MBq/s/kg, resulting in a consistent total injected activity of 10 MBq/kg (shaded *blue area*) administered over a standard time interval of 30 seconds. The CF-mode profiles result in the same total activity, but injected over variable time intervals from 10 to 30 s and with variable peak amplitudes from 0.4 to 2.2 MBq/s/kg

Depending on the amount of ^82^Rb activity available from the ^82^Sr/^82^Rb generator and the amount of requested activity, the CF-mode of elution resulted in variable time intervals and peak amplitudes of tracer activity, as illustrated in Figure 3. In contrast, the CA-mode elution profiles were much less variable in shape, with very consistent weight-adjusted amplitude over the time course of infusion.

### Image Quality

**Figure 4:**
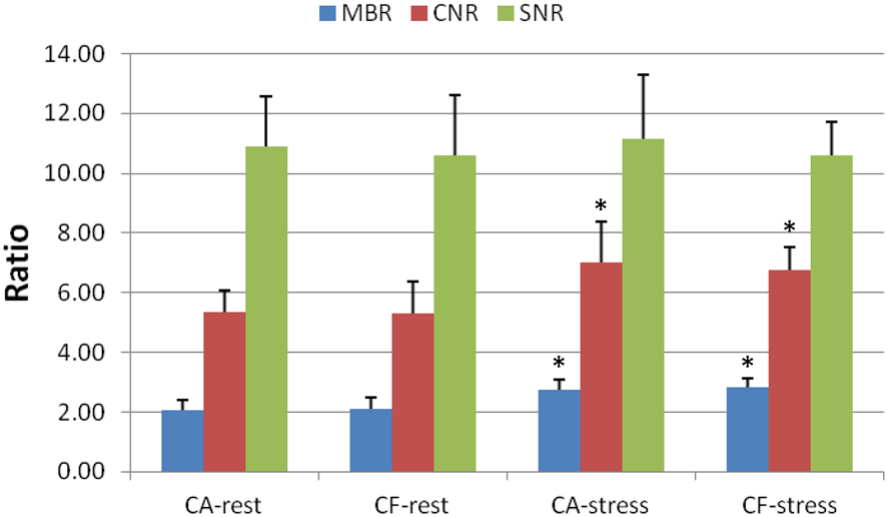
Myocardial perfusion image quality in the CA–CF cohort (mean + SD). There were no significant differences in myocardium-to-blood ratio (MBR), contrast-to-noise ratio (CNR), and myocardial signal-to-noise ratio (SNR) between CA vs CF infusion modes at rest or stress. There was a significant improvement in MBR and CNR at stress vs rest, as expected, using both infusion modes. *P < 0.05 increased vs rest

**Figure 5:**
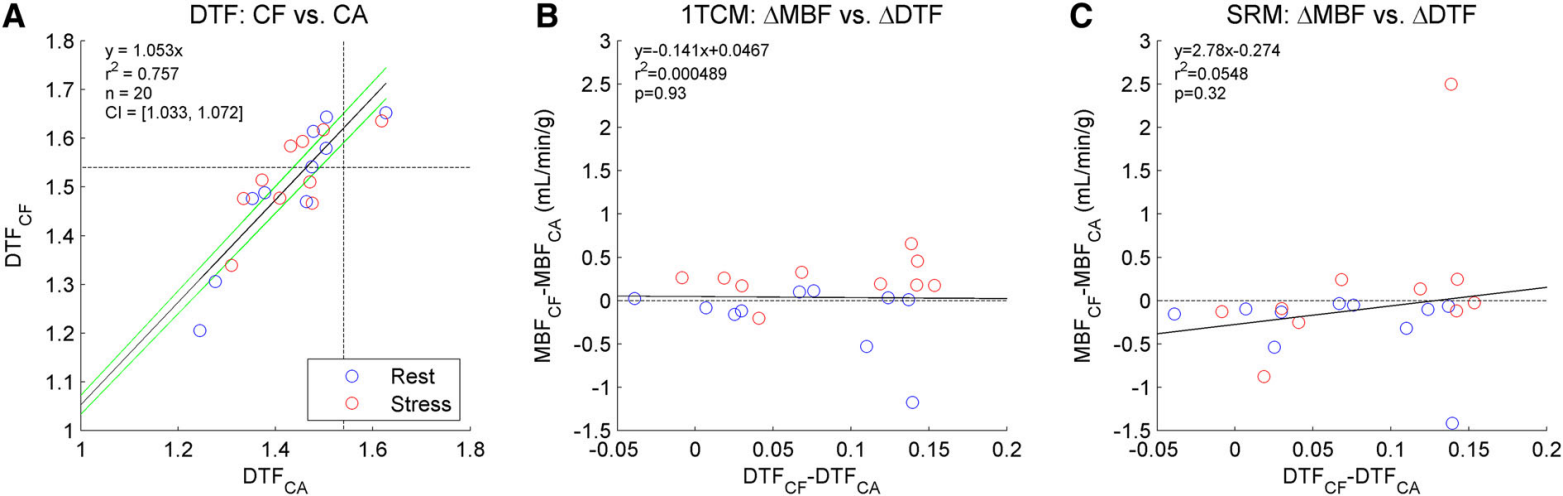
System dead-time factors (DTF) effect on myocardial blood flow (MBF). (**A**) Correlation of DTF values for paired rest (*blue*) and stress (*red*) scans using constant activity (CA) and constant flow (CF) infusions. *Dotted lines* are shown at the DTF value of 1.54, corresponding to the maximum recommended dead-time limit of 35%. Differences in DTF between infusion types did not correlate with changes in MBF, using either the 1TCM (**B**) or SRM (**C**) analysis method

Comparison of the image quality metrics between the CA vs CF infusions in the CA–CF cohort (*n* = 10) is summarized in Figure 4. The CA ‘square-wave’ infusions had significantly lower peak DTF values compared to the CF ‘bolus’ infusions (1.43 ± 0.10 vs 1.51 ± 0.12; P < 0.001). A larger proportion of CF scans was above the target dead-time factor (1.54) and correction inaccuracy recommended on the particular PET scanner used in this study (8/20 vs 2/20, *P* = 0.01) as shown in Figure 5A; likely due to the higher injected activity rates as shown in Figure 3. The total coincidence (prompt) counts recorded in the uptake phase were >10% higher (69 vs 62 M; *P* < 0.001) using the CA vs CF infusion mode. Despite this small increase in recorded counts, there was no significant difference observed in the uptake image quality metrics including MBR, CNR, and SNR between CA and CF infusion modes (Figure 4).

### Myocardial Blood Flow

**Figure 6:**
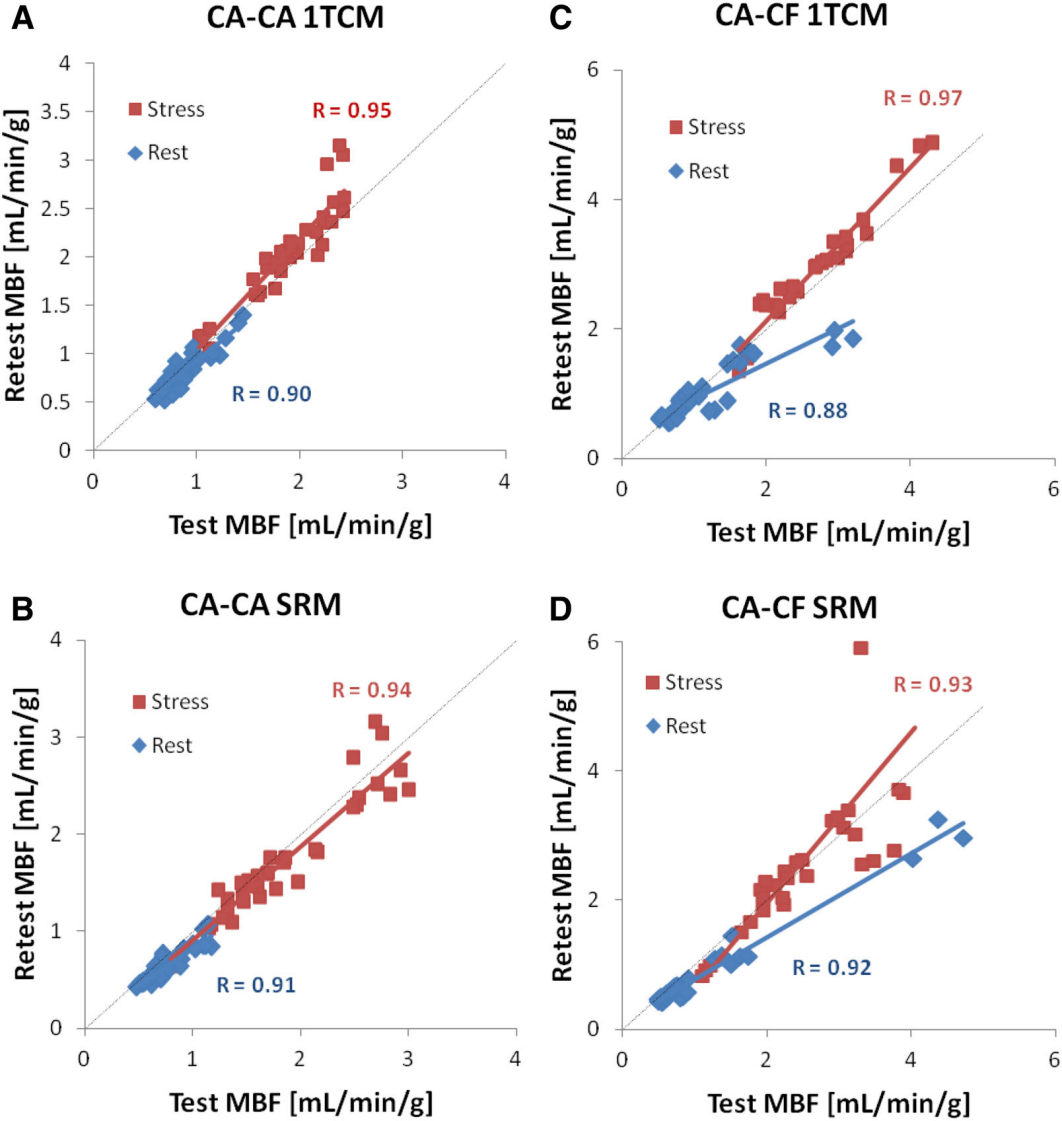
Retest vs test MBF values at rest and stress using the single-tissue compartment model (1TCM) and the simplified retention model (SRM)

Test–retest MBF scatter plots are shown in Figure 6 using consistent (CA–CA) and variable (CA–CF) infusion profiles, as analyzed using the 1TCM and SRM tracer kinetic methods. The CA–CA cohort (Figure 6A, B) generally displayed test–retest values closer to the line of identity (ideal) compared to the CA–CF values (Figure 6C, D), using both the 1TCM and SRM methods. The measured range of MBF values was wider at rest and stress in the CA–CF vs CA–CA group, due to several outliers apparent in the CA–CF scatter plots, using both the 1TCM and SRM methods.

**Figure 7:**
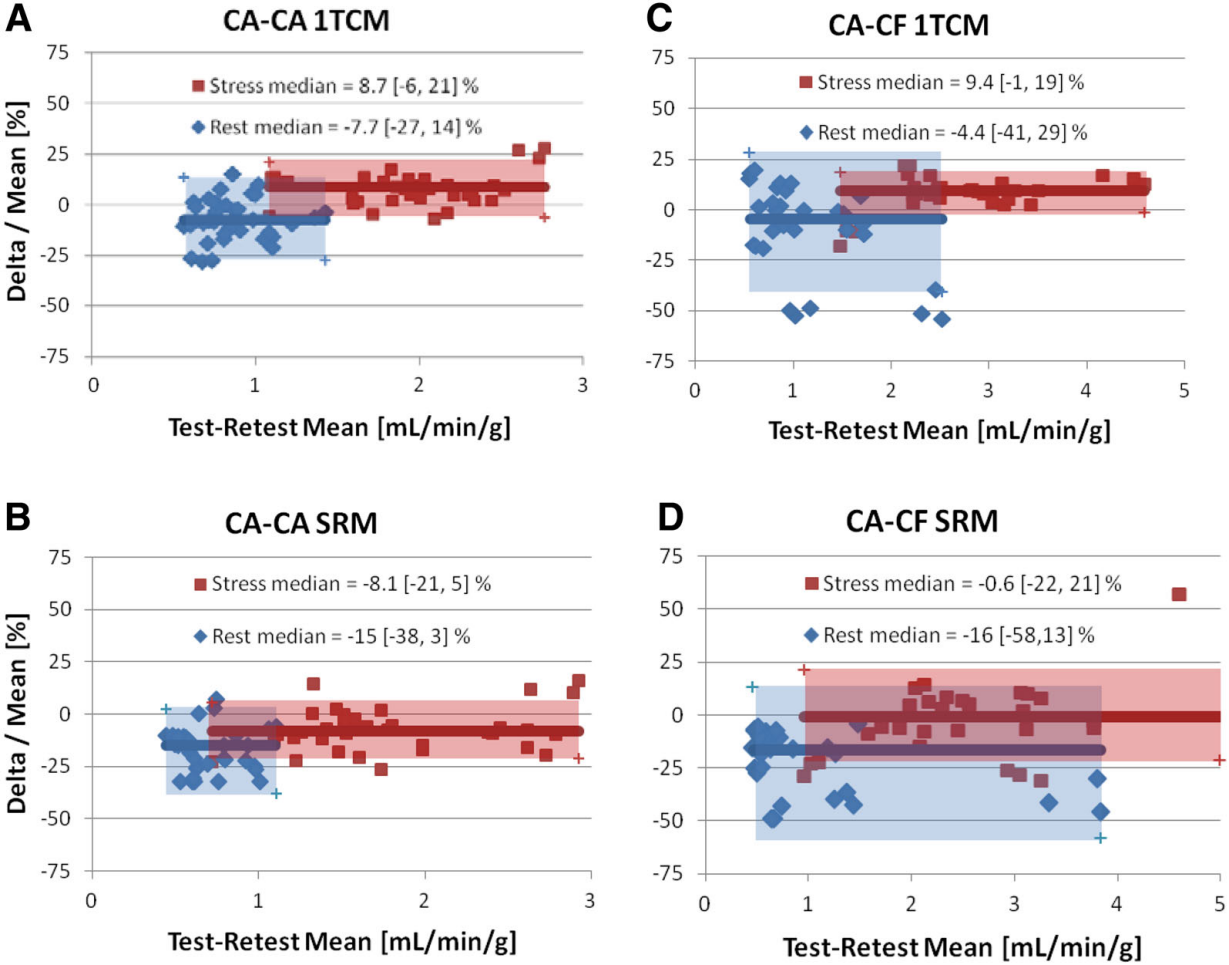
Bland–Altman plots of MBF repeatability at rest and stress. Retest–test delta/mean [%] values are plotted at rest (*blue*) and stress (*red*) vs the respective Mean MBF [mL/min/g]. Median values are plotted as *thick solid lines* within the shaded regions illustrating the limits of agreement of the median ± 1.45 × IQR (inter-quartile range). Values are indicated as median and [lower, upper] limits of agreement

The corresponding Bland–Altman plots of the relative test–retest Delta/Mean [%] values are shown in Figure 7, together with the 95% limits of agreement (median ± RPC_np_). The subjects with consistent tracer infusion profiles (CA–CA cohort) generally had a smaller range of mean MBF values at rest and stress, with narrower limits of agreement (Figure 7A, B), indicating improved test–retest repeatability.

The full list of test–retest median and RPC_np_ values at Stress and Rest, as well as the combined Stress & Rest, stress/rest, and stress–rest reserve values are shown in Table 3; expressed both as absolute (delta) and relative (delta/mean [%]) differences in MBF. Significantly better test–retest variability (lower RPC_np_) was measured in the CA–CA vs CA–CF cohort at rest, stress & rest combined, stress/rest, and stress–rest reserve, using both the 1TCM and SRM analysis methods (*P* < 0.05). The test–retest variability was also significantly lower at stress in the CA–CA vs CA–CF cohort using the SRM analysis method.

**Table 3:** Test–retest MBF repeatability statistics

	Cohort	Model	Spearman *R*	Median delta (delta/mean %)	RPC_np_ (IQR × 1.45) delta (delta/mean %)
Stress (mL/min/g)	CA–CA	1TCM	0.95	0.16 (8.7)^‡^	0.25 (14)^§^
		SRM	0.94	−0.13 (−8.1)^‡^	0.27 (15)*
	CA–CF	1TCM	0.97	0.26 (9.4)^‡^	0.32 (11)
		SRM	0.93	−0.02 (−0.6)	0.68 (25)
Rest (mL/min/g)	CA–CA	1TCM	0.90	−0.06 (−7.7)^‡^	0.18 (21)*
		SRM	0.91	−0.10 (−15)^‡^	0.16 (21)*
	CA–CF	1TCM	0.88	−0.05 (−4.4)^‡^	0.32 (36)
		SRM	0.92	−0.11 (−16)^‡^	0.37 (38)
Stress & rest (mL/min/g)	CA–CA	1TCM	0.98	0^†^	0.21 (16)*
		SRM	0.98	0^†^	0.17 (22)*
	CA–CF	1TCM	0.94	0^†^	0.34 (19)
		SRM	0.97	0^†^	0.42 (38)
Stress/rest (ratio)	CA–CA	1TCM	0.87	0.07 (3.3)	0.40 (20)*^§^
		SRM	0.87	−0.55 (−25)^‡^	0.74 (23)*
	CA–CF	1TCM	0.74	0.03 (1.6)	0.66 (27)
		SRM	0.86	−0.61 (−17)^‡^	1.75 (49)
Stress–rest (mL/min/g)	CA–CA	1TCM	0.93	0.09 (8.9)^‡^	0.22 (24)*
		SRM	0.91	−0.27 (−23)^‡^	0.26 (29)*
	CA–CF	1TCM	0.77	0.13 (8.4)	0.49 (34)
		SRM	0.53	−0.15 (−7.1)	0.79 (68)

Delta = retest−test; mean = (test + retest)/2

^‡^ *P* < 0.05 significant bias in the median delta vs zero

^†^ Adjusted for the median rest and stress delta values

^§^ Lowest values for combined interpretation of stress and stress/rest MBF

* *P* < 0.05 decreased variance in CA–CA vs CA–CF cohort

**Figure 8:**
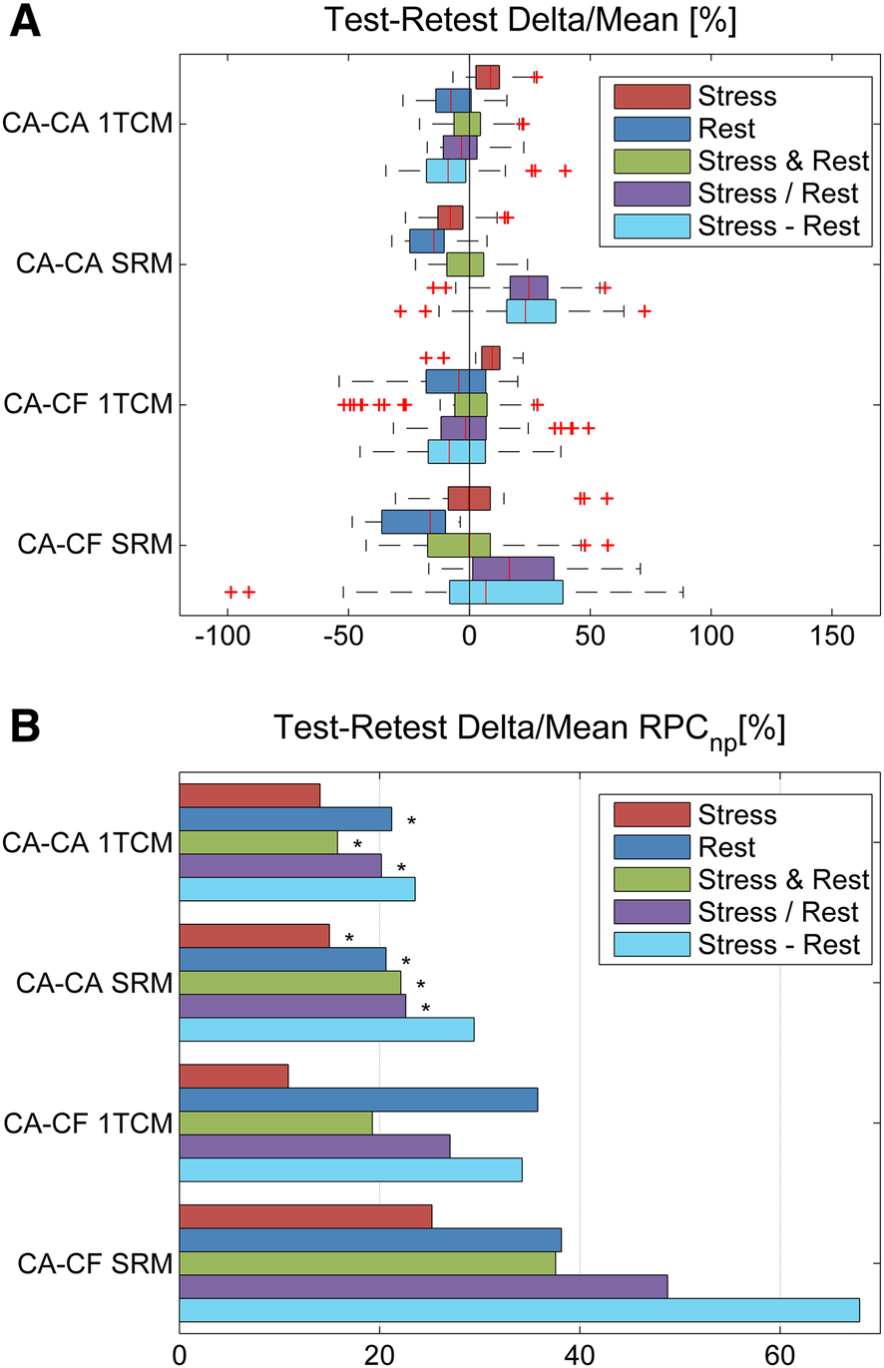
Test–retest RPC_np_ values of delta MBF [%] relative to the mean. (**A**) Box-plots of the median and inter-quartile range in the CA–CA and CA–CF cohorts, measured using the 1TCM and SRM methods; possible outliers shown with red ‘+’ symbols are beyond the median ± 1.5 × IQR. (**B**) Repeatability coefficients (RPC_np_) in the CA–CA and CA–CF cohorts, measured using the 1TCM and SRM methods. *P < 0.05 decreased variance in CA–CA vs CA–CF cohort

Box-plots (median and IQR) and repeatability coefficients (RPC) of the relative Delta/Mean [%] values are illustrated in Figures 8A, B, respectively, for stress, rest, stress & rest combined, stress/rest, and stress–rest reserve. It is apparent that the CA–CA cohort analyzed using the 1TCM method generally displayed the smallest median delta and RPC_np_ values, as well as the fewest outliers, whereas the CA–CF cohort analyzed using the SRM method had the largest RPC_np_ values at Stress, Rest, Stress & Rest combined, Stress/Rest, and stress–rest reserve, as well as the largest outliers. Similar patterns were observed in the RPC_np_ values using the absolute scale differences, as shown in the Supplementary Figure S1.

While the peak DTF values were approximately 5% higher using CF compared to the corresponding CA-mode values (Figure 5A), there were no significant differences in MBF observed as a result of changing DTF values using either the 1TCM or SRM (Figure 5B, C, respectively). The single outlier at stress observed using the SRM (Figures 5C, 6D) did not appear as an outlier using the 1TCM (Figures 5B, 6C), and therefore cannot be attributed to changes in dead-time alone.

## Discussion

To the best of our knowledge, this is the first study to evaluate the impact of tracer administration profiles on the test–retest repeatability of MBF quantification using dynamic ^82^Rb PET imaging. This work demonstrates that the ^82^Rb tracer infusion profile can be a significant source of variability in measured MBF values, and that precision can be improved using the more consistent CA infusion mode. Variability in the combined Stress&Rest MBF and Reserve measurements was substantially lower on average (20% vs 27% RPC_np_) when using consistent infusions (CA–CA) compared to variable infusion profiles (CA–CF), with the 1TCM analysis method (Table 3). This improvement in repeatability was even more pronounced when using the SRM kinetic analysis method (25% vs 52% RPC_np_), which appears to be more sensitive to changes in the shape of the infusion profile than the 1TCM approach. The widely used 1TCM appears to be more robust to variations in the infusion profile than the simple retention model, but neither model fully corrected for unexpected changes in the shape of the arterial blood input function. In theory, the quantitative MBF results should not depend on the shape of the input function, because the compartment model is formulated to predict the myocardial tissue response curve for any arbitrary shape of input. However, our results show that in practice, there is still some residual bias which can be reduced by standardizing the shape of the blood input function. Conversely, the simplified retention model does not predict the myocardial tissue response curve explicitly, and is therefore more reliant on a consistent shape of input function to provide repeatable measurements of MBF. While the repeatability of MBF quantification improved with consistent infusion profiles, it is equally important to note that the quality of the late-phase uptake images was not adversely affected using longer infusions.

Using the Rb-82 elution system that delivers reproducible ‘square-wave’ infusion profiles over the life of the generator, our results suggest that the CA infusion mode is preferable for optimal repeatability, in particular when using the SRM for MBF quantification with ^82^Rb PET. This finding is of clinical significance since non-invasive MBF and flow reserve imaging is being used increasingly as part of the routine clinical evaluation of stress myocardial perfusion. The results of this study indicate that the repeatability of MBF measurements with ^82^Rb PET is improved using an elution system capable of maintaining reproducible infusion profiles irrespective of generator age.

### MBF Tracer Kinetic Models

We evaluated the repeatability of MBF quantification using two commonly employed methods: the single-tissue-compartment kinetic model (1TCM) and a simplified retention model (SRM). The 1TCM is widely accepted in the community to be both accurate and robust.^1^,^2^,^6^ Previous studies have shown good reproducibility of the 1TCM for ^82^Rb MBF measurement, as implemented in several research and commercial software packages.^6^,^23^ Simplified retention models may be attractive due to the lower computational complexity but suffer from relatively poor reproducibility among results reported by various laboratories.^17^ For example, despite the widespread acceptance of ^82^Rb and ^13^N-ammonia PET imaging for the quantification of MBF, quite a wide range of tracer retention fractions have been reported (20 to 30% for ^82^Rb and 30 to 60% for ^13^N-ammonia at peak stress) using several variations of a simple retention model, each with slightly different underlying assumptions and numerical implementations.^17^

To date, there has been no study comparing the 1TCM vs SRM methods, in terms of MBF test–retest repeatability. Although it was not the primary objective of the present study, we observed substantially lower test–retest variability using the 1TCM compared to SRM in the CA–CF cohort, for the combined Stress&Rest MBF and Reserve data (27% vs 52% RPC_np_). However, when using consistent infusion profiles (CA–CA), the differences in repeatability between the 1TCM vs SRM were smaller (20% vs 25% RPC_np_) and did not reach statistical significance. These findings support our hypothesis that changes in the tracer infusion profile shapes can adversely impact MBF variability and that a reproducible infusion profile can help to reduce test–retest variability, especially when assuming SRM kinetics.

### Outlier Values

The regression and Bland–Altman plots in Figures 6 and 7 suggest that one of the patients in the CA–CF cohort appears to be a clear outlier. This is particularly evident using the SRM method which resulted in abnormally high mean MBF values of 3 to 4 mL/min/g at rest, and up to 6 mL/min/g at stress. Several quality assurance metrics were investigated to identify a potential cause; there was no significant patient body motion or hemodynamic changes between test and retest scans. The tracer elution profiles, had substantially different shapes between the CA ‘square-wave’ vs CF ‘bolus’ infusion modes, but were highly repeatable between rest and stress as shown in Supplementary Figure S2. The corresponding blood input and myocardium TACs for this particular patient are presented in Supplementary Figure S3. The initial rest scan had a delayed rise in blood activity following the initial bolus, which may be associated with an interstitial infusion or partially blocked IV catheter. The second rest and both stress scans had a similar delayed but smaller-amplitude blood peak at approximately 1.5 min following the initial bolus. The shape of the blood input curves was reflected in abnormally long mean-transit-times, as derived using a gamma-variate curve fit to the first-pass blood peak activity. Consequently, tracer delivery to the myocardium proceeded well beyond the standard blood integration time of the SRM, leading to substantial overestimation of the MBF values (>4 mL/min/g) during the initial test scan at rest. In contrast, the 1TCM was better able to account for the complete shape of the blood and myocardium TACs, resulting in more repeatable MBF estimates despite the dramatic changes in shape. These results suggest an improved ability of the 1TCM to accommodate a wider range of arterial blood and myocardium TAC shapes that may be encountered in clinical practice.

### Comparison to Previous Studies

Table 4 summarizes previously published repeatability values for stress flow, rest flow, and flow reserve using PET, compared with the results of the current study. The values reported in this study are among the lowest, which may benefit from our previous work on minimizing several sources of variability including: kinetic model parameters, image-derived blood input ROI,^10^ and operator variability.^5^ In the current study, we used these previously determined optimal parameters and investigated the infusion profile shape as an additional source of variability. The present results demonstrate that ^82^Rb PET MBF quantification using the CA elution mode to deliver consistently shaped infusion profiles can improve test–retest precision.

**Table 4:** Test–retest MBF repeatability values reported in the literature

Author	Tracer	Retest interval	Stressor	Stress RPC (mL/min/g)	Rest RPC (mL/min/g)	Stress/rest RPC (ratio)
Kaufmann^24^	^15^O-water	10 minutes	Adenosine	0.90 (25%)	0.17 (18%)	0.98 (34%)
Wyss^25^	^15^O-water	20 minutes	Adenosine	1.34 (27%)	0.26 (21%)	1.44 (35%)
Siegrist^26^	^15^O-water	40 minutes	Cold-Pressor	1.82 (NA)	1.47 (NA)	NA
Schindler^27^	^13^N-ammonia	45 minutes	Cold-Pressor	0.28 (32%)	0.26 (39%)	0.27 (23%)^†^
Nagamachi^28^	^13^N-ammonia	50 minutes	Adenosine	0.40 (20%)	0.13 (20%)	NA
Manabe^29^	^82^Rb-chloride	60 minutes	Adenosine Triphosphate	0.92 (27%)	0.19 (24%)	1.61 (36%)
Sdringola^30^*	^82^Rb-chloride	22 days	Dipyridamole	1.09 (41%)	0.24 (35%)	1.96 (51%)
Efseaff^10^	^82^Rb-chloride	15 minutes	Dipyridamole	NA	0.21 (25%)	0.58 (24%)**
Moody^13^***	^82^Rb-chloride	NA	Regadenoson	0.51 (28%)	0.28 (26%)	NA
Johnson^12^	^82^Rb-chloride	NA	Dipyridamole	0.76 (34%)	0.33 (39%)	0.94 (34%)
Klein^§^	^82^Rb-chloride CA–CF	10 minutes	Dipyridamole	0.32 (11%)	0.32 (36%)	0.66 (27%)
	^82^Rb-chloride CA–CA	10 minutes	Dipyridamole	0.25 (14%)	0.18 (21%)	0.40 (20%)

*NA* not available

^†^ Estimated from reported RPC of S-R difference

* 2-Week test–retest interval in ‘not normal’ cohort

** Estimated using test–retest variance at rest only

*** predicted ‘short-term’ RPC using analytical variance estimation in clinical patient scans

^§^ Using 1TCM analysis method in the present study

This work was performed using a high-count-rate LYSO-based PET system. High-count-rate capabilities are essential for MBF quantification with ^82^Rb due to the wide range of count rates encountered over the course of dynamic image acquisition, associated with rapid tracer distribution and short radioactive half-life (76 s). This is especially true in a clinical setting where high-quality MPI (and ECG-gated) images are desired in addition to dynamic imaging for MBF quantification using a single tracer injection. CA infusions may prove to be even more advantageous on lower count-rate systems (e.g., using BGO detectors) in which a tradeoff exists between higher ^82^Rb activity required for diagnostic quality MPI images, vs lower ^82^Rb activity to avoid detector dead-time saturation in early time frames for accurate MBF quantification.

### Potential Limitations

This study enrolled a relatively small number of subjects (*n* = 22). Nevertheless, we were able to demonstrate a significant improvement in MBF repeatability using a consistent CA-mode infusion of ^82^Rb compared to variable CF-mode infusions, which demonstrated changes in amplitude and duration as a function of generator age and injected activity.

The present study did not investigate the test–retest repeatability of the CF-mode infusions alone, with variable shape profiles at the start and end of the generator shelf-life, as may be encountered in clinical practice. Our results suggest that the CA ‘square-wave’ infusion mode might be expected to improve MBF repeatability compared to CF-mode studies performed on different days, by removing the variations between infusion profiles that typically occur as the generator ages. This could be confirmed in a future study using CA–CA vs CF–CF test–retest studies acquired on different days within the normal generator shelf-life. In the present study, we were bound by the need to comply with our clinical-standard procedure for cardiac imaging and MBF quantification using the established CA-mode infusion, and selected a rapid test–retest protocol to achieve stable hemodynamics, which could otherwise affect the test–retest repeatability. Furthermore, the protocol was limited to 4 scans per subject in order to reduce participant discomfort and radiation exposure (estimated to be an additional 1.5 mSv above the clinical routine).^30^

Small differences in patient demographics between the CA–CA and CA–CF cohorts (Table 1) were an unintended consequence of our randomization strategy. Nevertheless, we believe that the conclusions remain valid since we evaluated the test–retest differences relative to the mean for each subject, and using paired comparisons in which each patient served as their own control.

## Conclusions

Myocardial blood flow quantification with ^82^Rb PET can be influenced by the shape of the time-activity infusion profile of the tracer. Constant-activity-rate ‘square-wave’ infusion of ^82^Rb produces more consistent activity profiles and improves the test–retest variability of MBF measurements, when compared to constant-flow-rate ‘bolus’ administration of ^82^Rb, especially using the simplified retention model. Standard MPI uptake-phase image quality was not influenced by the variations in tracer infusion profiles.

## New Knowledge Gained

The reproducibility of myocardial blood flow quantification from ^82^Rb dynamic PET may be influenced by inconsistent tracer infusion profiles. The one-tissue kinetic model is more robust to variations in tracer infusion than the simplified retention model. Therefore, a reproducible infusion profile over the life of a ^82^Sr/^82^Rb generator such as constant activity rate (square-wave) is preferable, especially when using a simplified retention model. Conventional MPI quality is not degraded by CA infusions.

## Electronic supplementary material

Below is the link to the electronic supplementary material.

Supplementary material 1 (PPTX 1181 kb)

Supplementary material 2 (DOCX 1179 kb)

## Abbreviations

MBF: Myocardial blood flow
CA: Constant-activity-rate
CF: Constant-flow-rate
1TCM: 1-Tissue compartment model
SRM: Simplified retention model
DTF: Dead-time factor
RPC_np_: Reproducibility coefficient (non-parametric-based estimate)
MBR: Myocardium-to-blood ratio
CNR: Contrast-to-noise ratio
SNR: Signal-to-noise ratio
