# Supplementary material for: Consistent tracer administration profile improves test–retest repeatability of myocardial blood flow quantification with 82Rb dynamic PET imaging
Source: J Nucl Cardiol. 2016 Nov 1;25(3):929–41. doi: 10.1007/s12350-016-0698-6 (PMC5966478; doi:10.1007/s12350-016-0698-6)
Supplement: Supplementary file 1 — Supplementary material 1 (PPTX 1181 kb) [file 12350_2016_698_MOESM1_ESM.pptx]

## Slide 1
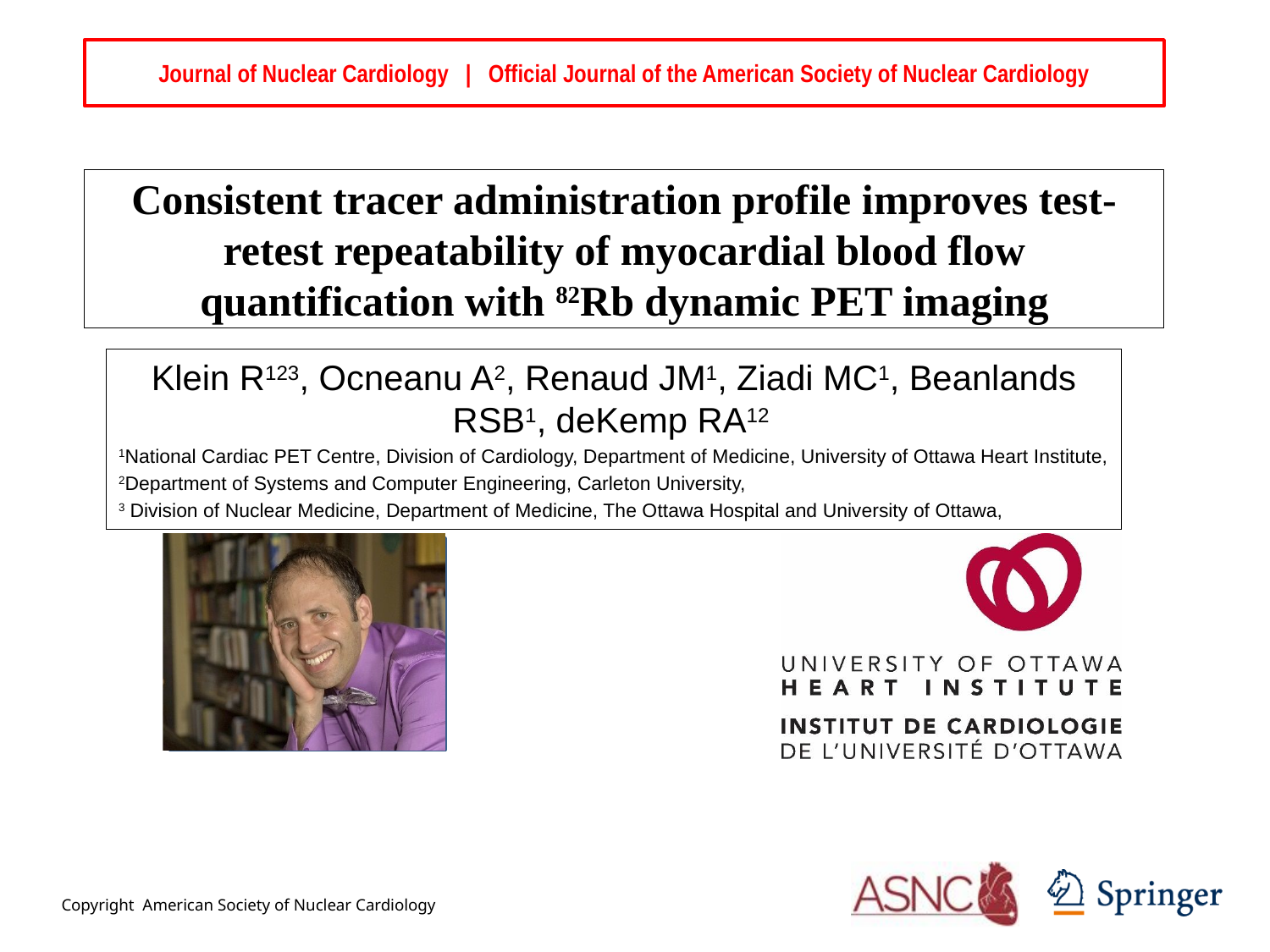

Journal of Nuclear Cardiology | Official Journal of the American Society of Nuclear Cardiology
# Consistent tracer administration profile improves test-retest repeatability of myocardial blood flow quantification with 82Rb dynamic PET imaging
Klein R123, Ocneanu A2, Renaud JM1, Ziadi MC1, Beanlands RSB1, deKemp RA12
1 National Cardiac PET Centre, Division of Cardiology, Department of Medicine, University of Ottawa Heart Institute,
2 Department of Systems and Computer Engineering, Carleton University,
3 Division of Nuclear Medicine, Department of Medicine, The Ottawa Hospital and University of Ottawa,
Head shot of author
required
Institution
Picture/Logo
Optional
Copyright American Society of Nuclear Cardiology

## Slide 2
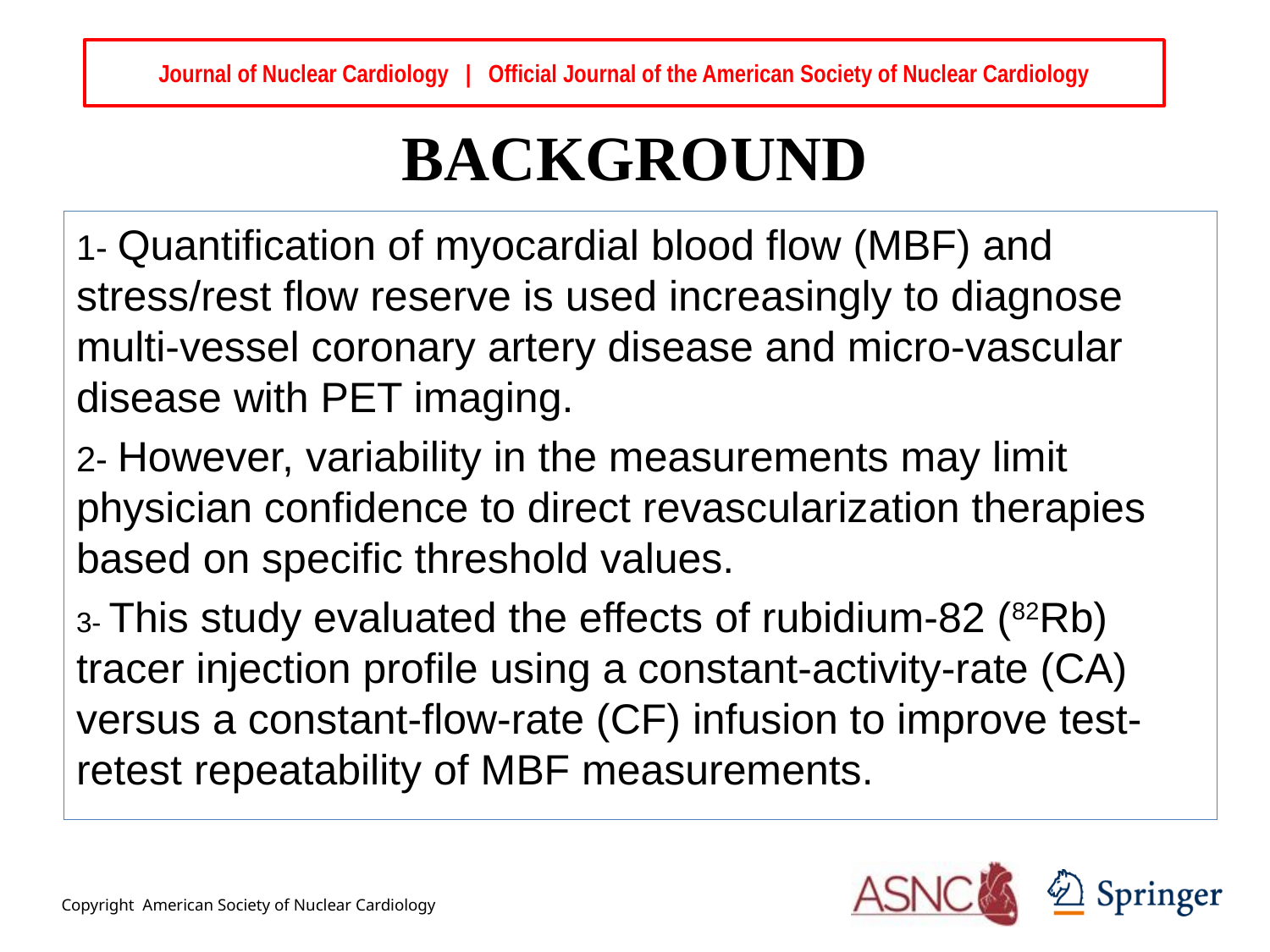

Journal of Nuclear Cardiology | Official Journal of the American Society of Nuclear Cardiology
# BACKGROUND
1- Quantification of myocardial blood flow (MBF) and stress/rest flow reserve is used increasingly to diagnose multi-vessel coronary artery disease and micro-vascular disease with PET imaging.
2- However, variability in the measurements may limit physician confidence to direct revascularization therapies based on specific threshold values.
3- This study evaluated the effects of rubidium-82 (82Rb) tracer injection profile using a constant-activity-rate (CA) versus a constant-flow-rate (CF) infusion to improve test-retest repeatability of MBF measurements.
Copyright American Society of Nuclear Cardiology

## Slide 3
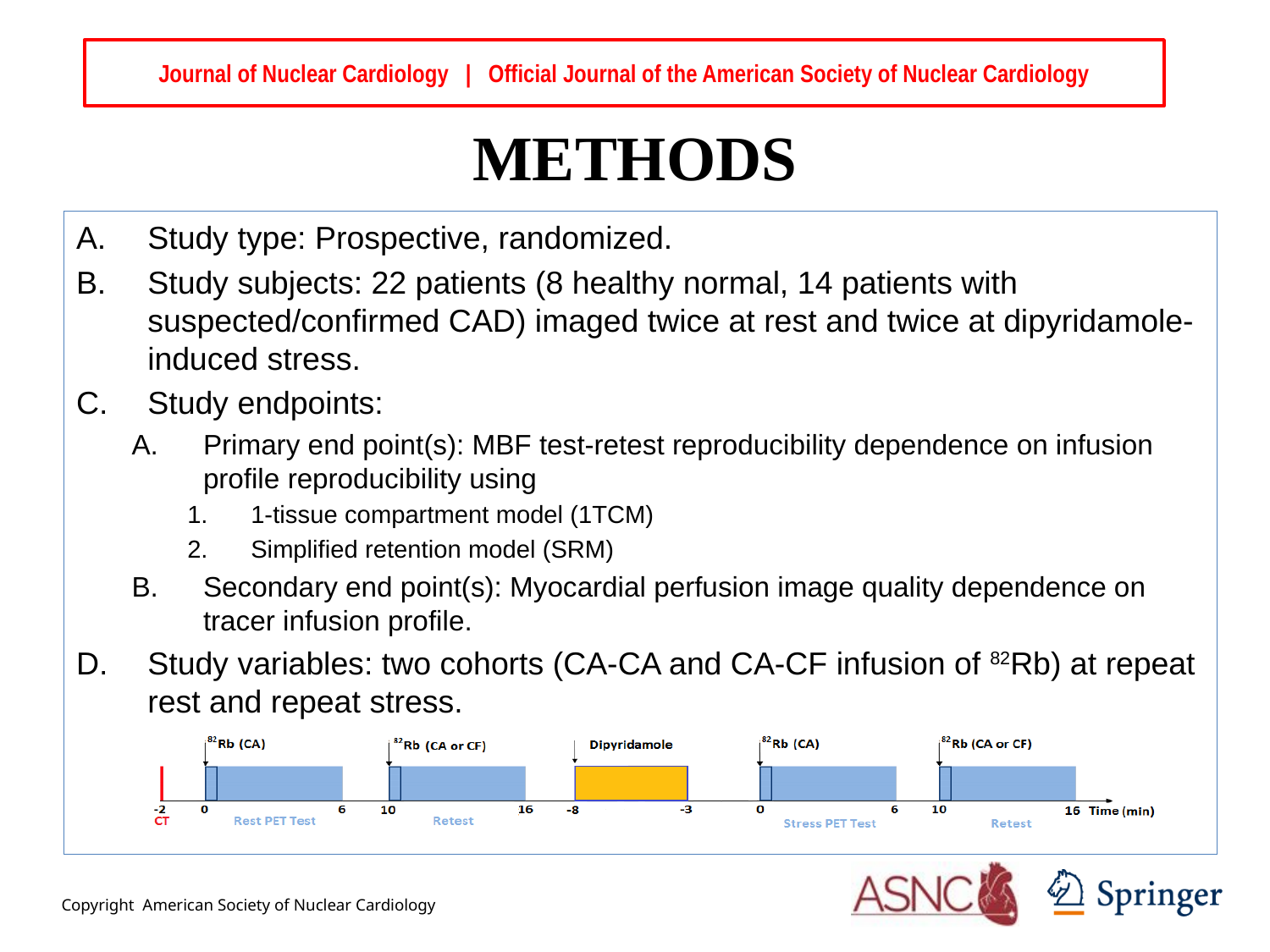

Journal of Nuclear Cardiology | Official Journal of the American Society of Nuclear Cardiology
# METHODS
Study type: Prospective, randomized.
Study subjects: 22 patients (8 healthy normal, 14 patients with suspected/confirmed CAD) imaged twice at rest and twice at dipyridamole-induced stress.
Study endpoints:
Primary end point(s): MBF test-retest reproducibility dependence on infusion profile reproducibility using
1-tissue compartment model (1TCM)
Simplified retention model (SRM)
Secondary end point(s): Myocardial perfusion image quality dependence on tracer infusion profile.
Study variables: two cohorts (CA-CA and CA-CF infusion of 82Rb) at repeat rest and repeat stress.
Copyright American Society of Nuclear Cardiology

## Slide 4
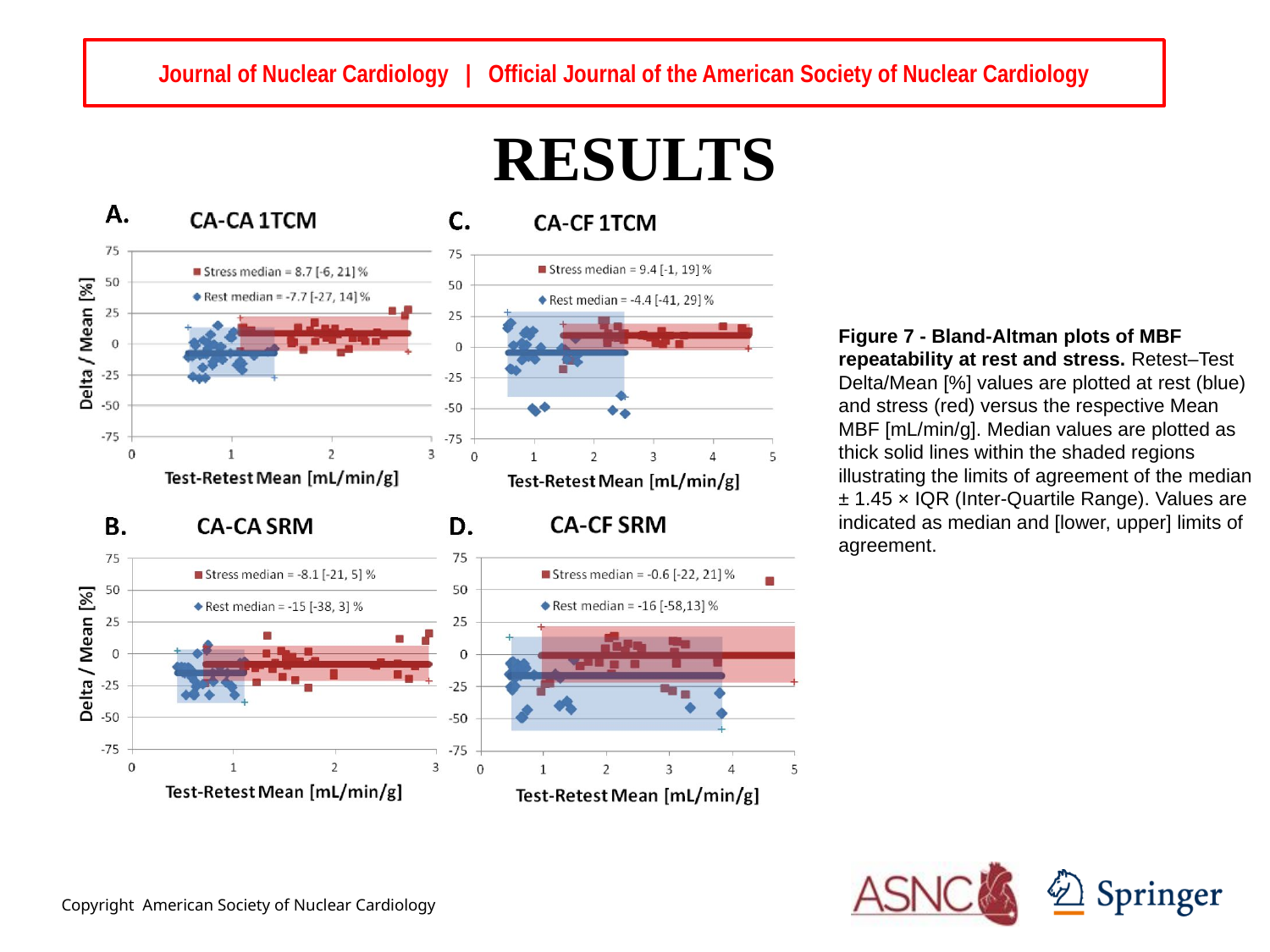

Journal of Nuclear Cardiology | Official Journal of the American Society of Nuclear Cardiology
# RESULTS
Figure 7 - Bland-Altman plots of MBF repeatability at rest and stress. Retest–Test Delta/Mean [%] values are plotted at rest (blue) and stress (red) versus the respective Mean MBF [mL/min/g]. Median values are plotted as thick solid lines within the shaded regions illustrating the limits of agreement of the median ± 1.45 × IQR (Inter-Quartile Range). Values are indicated as median and [lower, upper] limits of agreement.
Copyright American Society of Nuclear Cardiology

## Slide 5
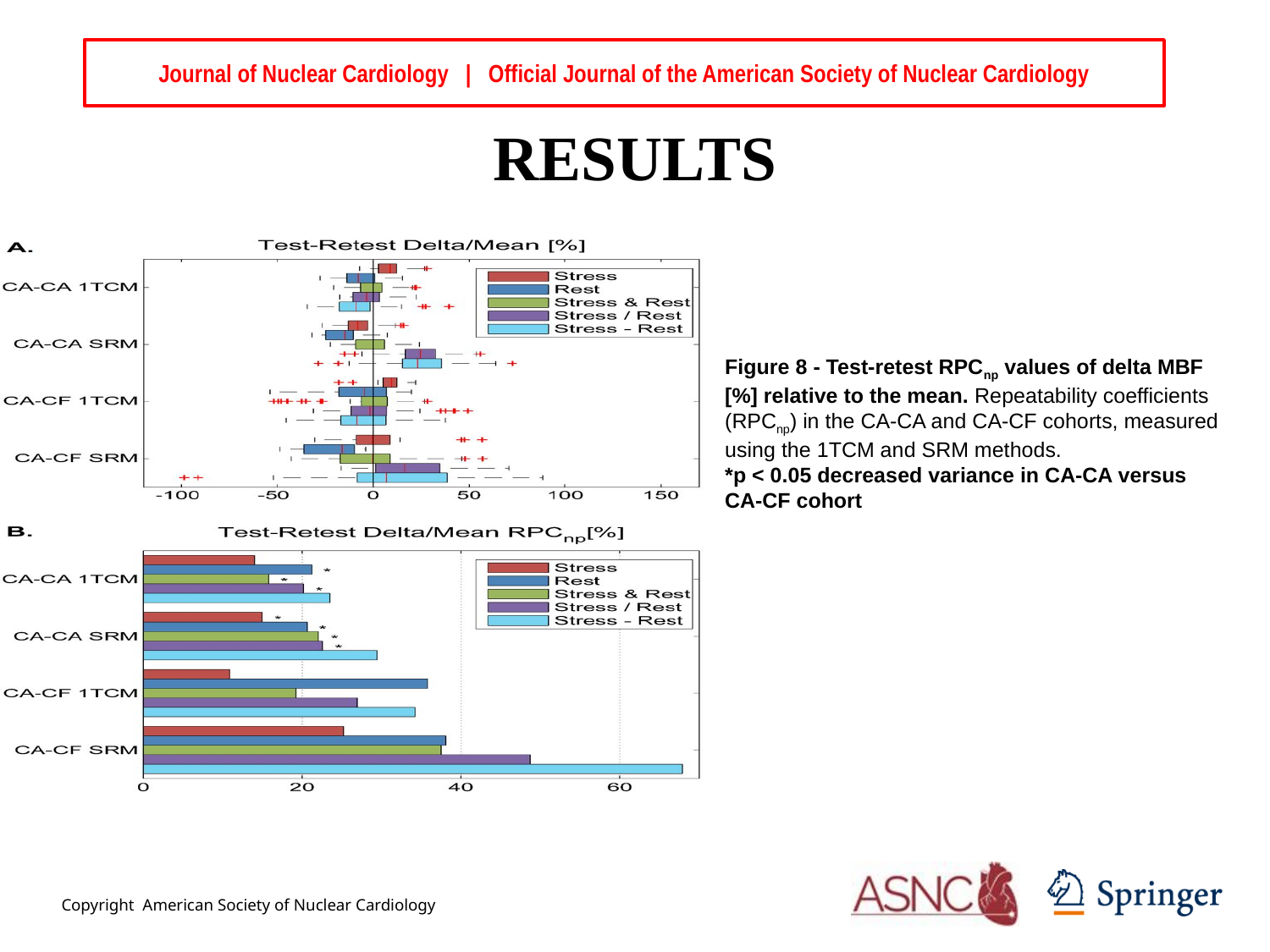

Journal of Nuclear Cardiology | Official Journal of the American Society of Nuclear Cardiology
# RESULTS
Figure 8 - Test-retest RPCnp values of delta MBF [%] relative to the mean. Repeatability coefficients (RPCnp) in the CA-CA and CA-CF cohorts, measured using the 1TCM and SRM methods.
*p < 0.05 decreased variance in CA-CA versus CA-CF cohort
Copyright American Society of Nuclear Cardiology

## Slide 6
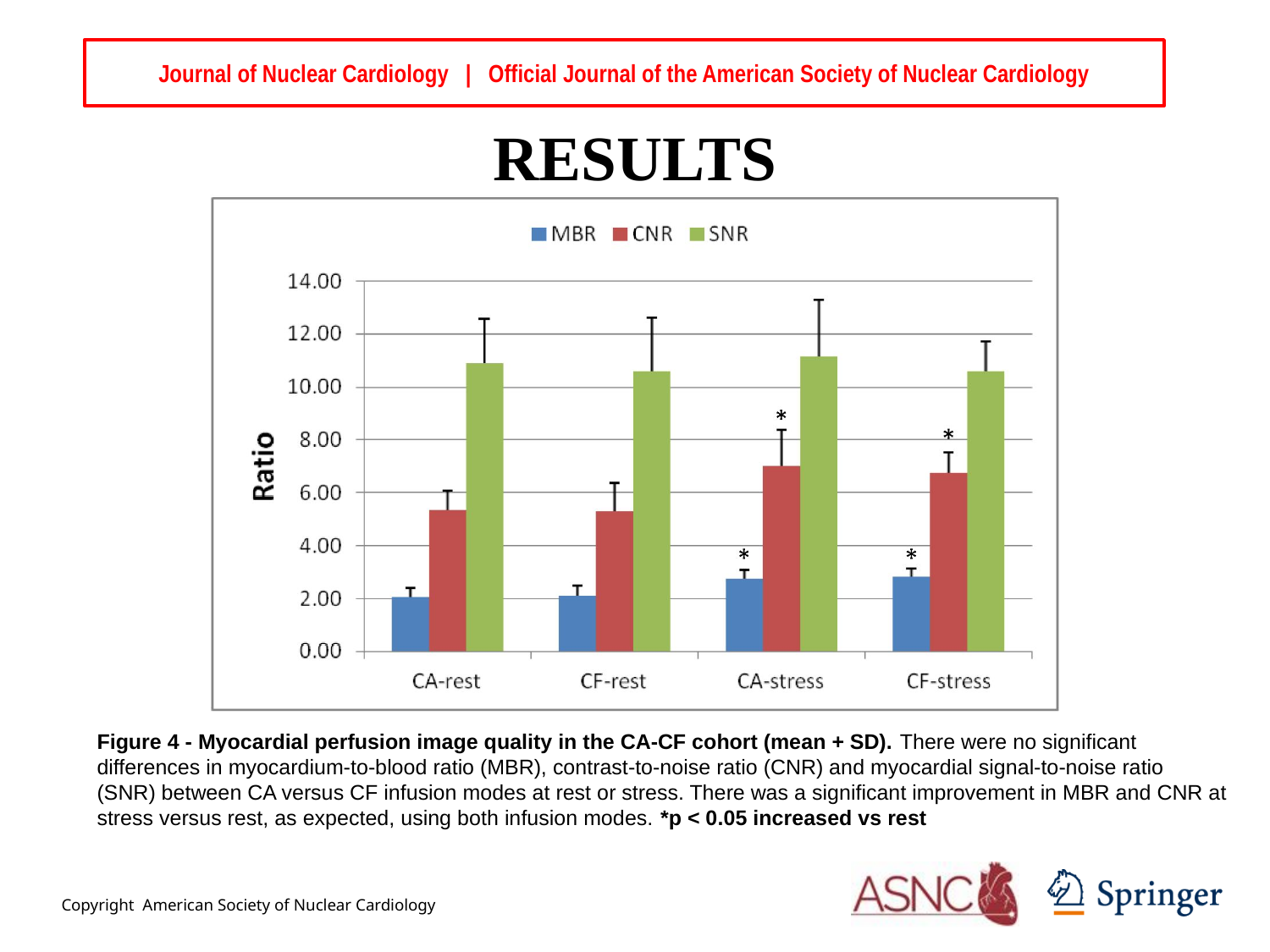

Journal of Nuclear Cardiology | Official Journal of the American Society of Nuclear Cardiology
# RESULTS
Figure 4 - Myocardial perfusion image quality in the CA-CF cohort (mean + SD). There were no significant differences in myocardium-to-blood ratio (MBR), contrast-to-noise ratio (CNR) and myocardial signal-to-noise ratio (SNR) between CA versus CF infusion modes at rest or stress. There was a significant improvement in MBR and CNR at stress versus rest, as expected, using both infusion modes. *p < 0.05 increased vs rest
Copyright American Society of Nuclear Cardiology

## Slide 7
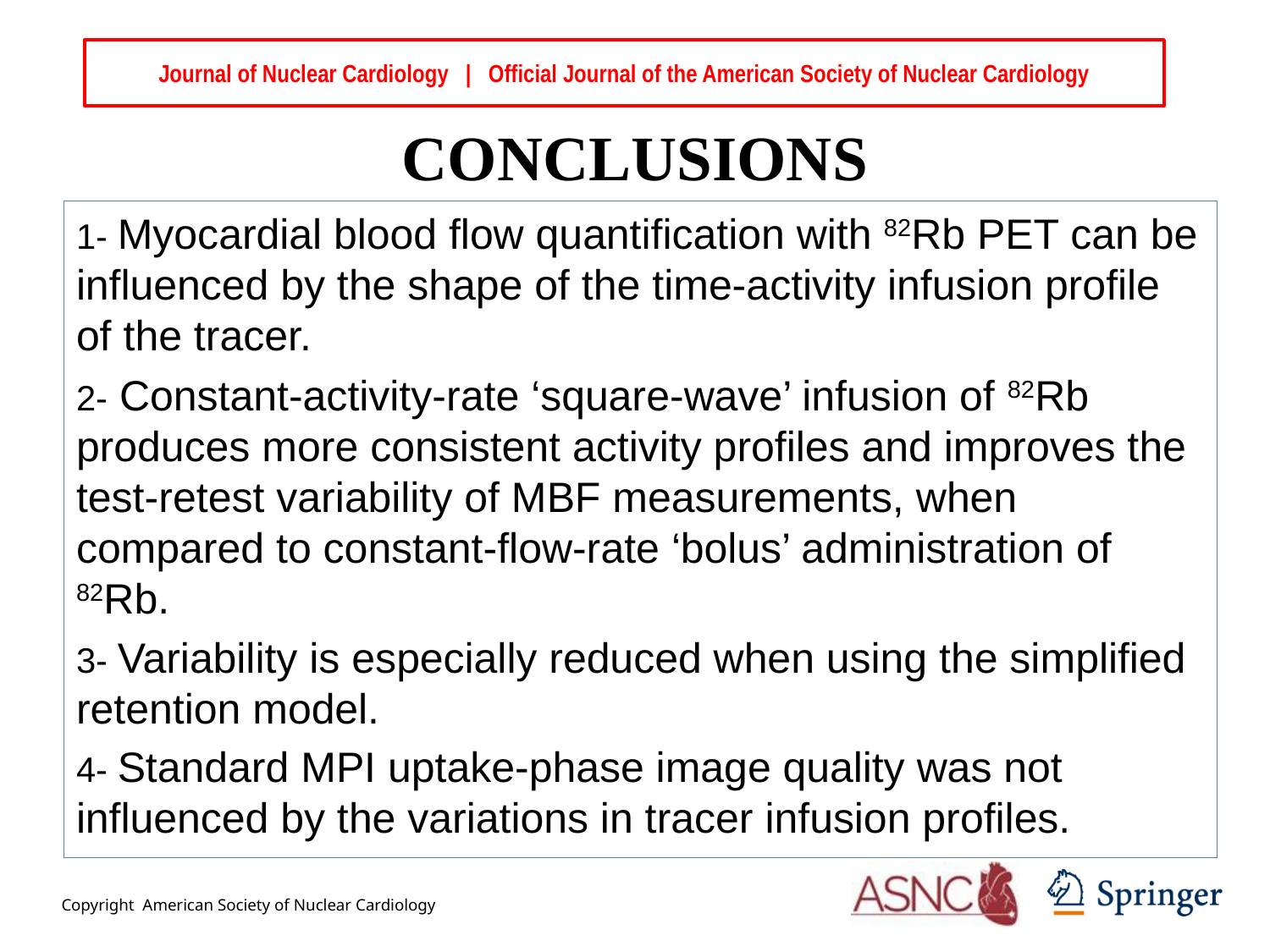

Journal of Nuclear Cardiology | Official Journal of the American Society of Nuclear Cardiology
# CONCLUSIONS
1- Myocardial blood flow quantification with 82Rb PET can be influenced by the shape of the time-activity infusion profile of the tracer.
2- Constant-activity-rate ‘square-wave’ infusion of 82Rb produces more consistent activity profiles and improves the test-retest variability of MBF measurements, when compared to constant-flow-rate ‘bolus’ administration of 82Rb.
3- Variability is especially reduced when using the simplified retention model.
4- Standard MPI uptake-phase image quality was not influenced by the variations in tracer infusion profiles.
Copyright American Society of Nuclear Cardiology
