# Supplementary material for: Consistent tracer administration profile improves test–retest repeatability of myocardial blood flow quantification with 82Rb dynamic PET imaging
Source: J Nucl Cardiol. 2016 Nov 1;25(3):929–41. doi: 10.1007/s12350-016-0698-6 (PMC5966478; doi:10.1007/s12350-016-0698-6)
Supplement: Supplementary file 2 — Supplementary material 2 (DOCX 1179 kb) [file 12350_2016_698_MOESM2_ESM.docx]

# Supplementary Figures





**Figure S1 Absolute scale MBF Test-Retest Delta values [mL/min/g]**. (A) Box-plots of the median and inter-quartile range in the CA-CA and CA-CF cohorts, measured using the 1TCM and SRM methods; possible outliers shown with red ‘+’ symbols are beyond the median ± 1.5xIQR. (B) Repeatability coefficients (RPCnp) in the CA-CA and CA-CF cohorts, measured using the 1TCM and SRM methods. Consistent infusion profiles in the CA-CA cohort resulted in more repeatable MBF and flow reserve values compared to the variable infusion profiles used in the CA-CF cohort. *p < 0.05 decreased values versus CA-CF


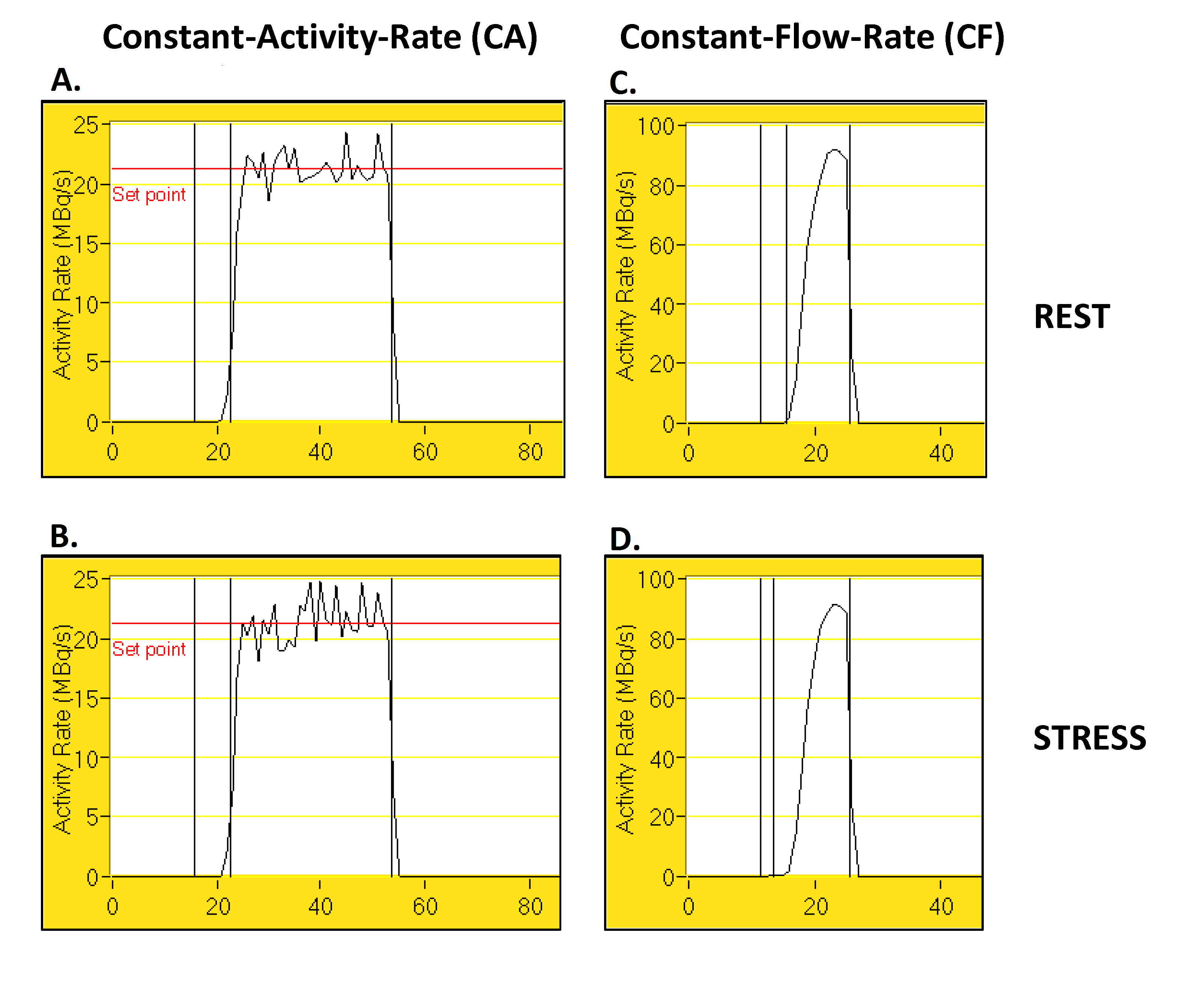


Figure S2 Rubidium Elution System (RBES) tracer injection profiles measured in the CA-CF outlier patient. (A) Rest CA, (B) Stress CA, (C) Rest CF, and (D) Stress CF elution profiles were highly repeatable at rest versus stress using the CA-mode (A vs. B) and CF-mode (C vs. D) infusions


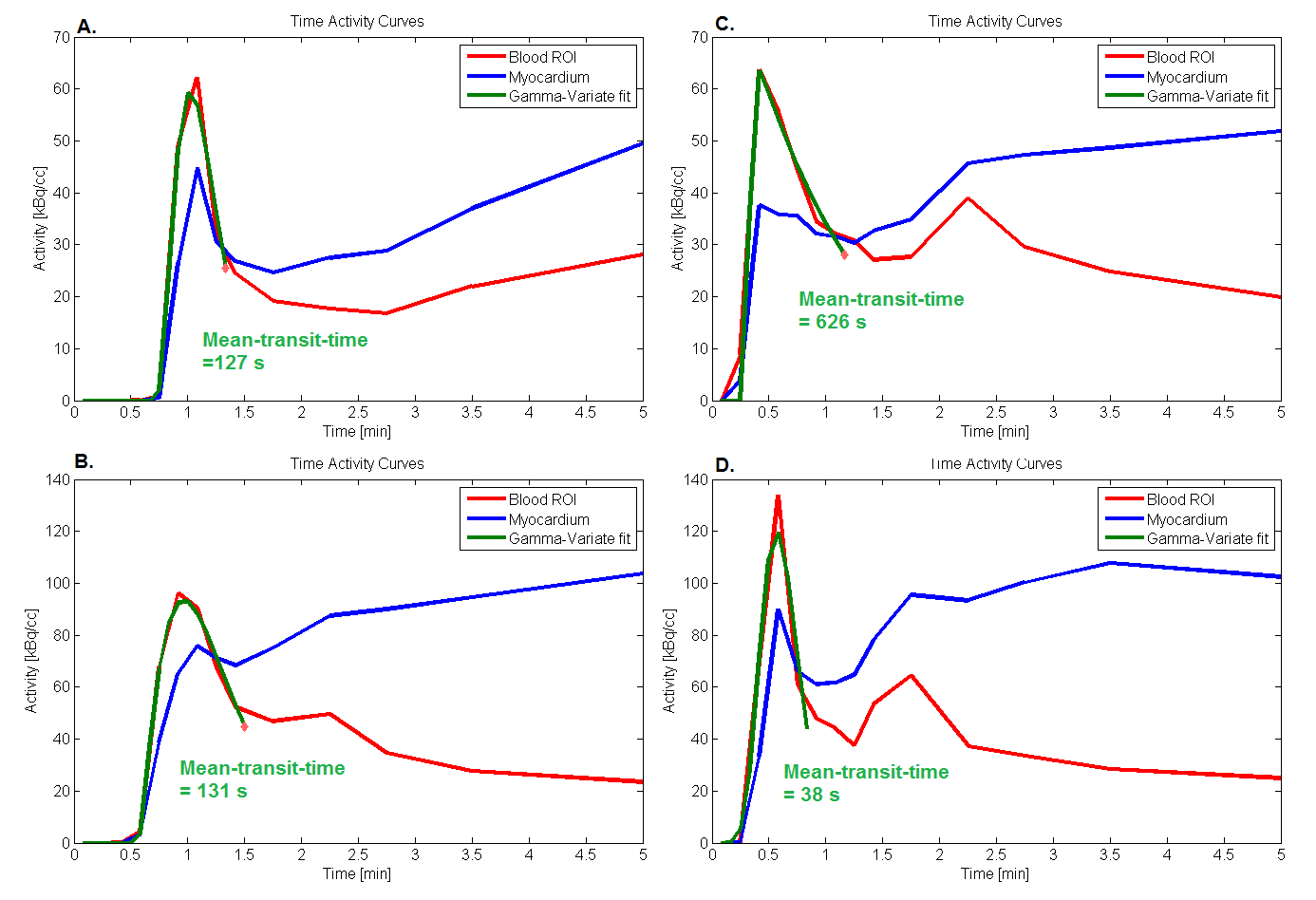


Figure S3 Time Activity Curves for the outlier patient in the CA-CF cohort. (A) Rest CA, (B) Rest CF, (C) Stress CA, and (D) Stress CF blood input and myocardium curves were highly variable in shape. The shape of the curves in (A) appears to be consistent with a partially interstitial injection, which continued to diffuse slowly back into the venous circulation following the initial infusion. With the CF-mode ‘bolus’ infusions (B ,D) there is an apparent second peak of radioactivity that appears at approximately 1.5 min following the initial bolus peak in the blood input function
